# Fate of subducted argon in the deep mantle

**DOI:** 10.1038/s41598-020-58252-8

**Published:** 2020-02-03

**Authors:** Shigeaki Ono

**Affiliations:** Volcanoes and Earth’s Interior Research Center, Research Institute for Marine Geodynamics, Japan Agency for Marine-Earth Science and Technology, 2-15 Natsushima-cho, Yokosuka, Kanagawa 237-0061 Japan

**Keywords:** Mineralogy, Structure of solids and liquids

## Abstract

The physical properties of argon (Ar) are investigated to 382 GPa and 3000 K using diamond anvil cell experiments and first-principles molecular dynamics. The estimated density of Ar is smaller that of the Preliminary reference Earth model (PREM) mantle, which indicates that the density crossover does not occur at the bottom of the lower mantle. A large volume dependence of the thermal pressure of Ar is revealed at pressures higher than 200 GPa, and a significant temperature dependence of the calculated effective Grüneisen parameters is confirmed at high pressures. A melting temperature of Ar is estimated from the calculation data and a significant pressure dependence is confirmed. If the pressure-temperature path of the subducted slab is lower than the critical condition, ~750 K and ~7.5 GPa, solid Ar can be carried down into the deep mantle. Melting of solid Ar in the upwelling mantle plume occurs at the bottom of the transition zone. Thus, solid Ar plays an important role in Ar recycling in the Earth’s interior.

## Introduction

It is known that noble gases are key tracers to understand the evolution of the Earth because of their inert nature and isotope variations. However, the mechanism of the recycling of noble gases in the deep mantle is still an open question. As the noble gases are the inert, any host phases of noble gases are not expected to transport into the deep mantle. Therefore, the role of grain boundaries in the storage of noble gases has been discussed^1,2^. Recently, an experimental study demonstrated high solubility of noble gases in amphibole^3^. Amphibole is commonly observed in the altered oceanic crust, with a significant amount of noble gases measured in natural rock samples from Ocean Drilling Program sites^4^. Amphibole has a mineralogical A-site, which is an energetically favourable position for noble gases. Lattice structures of some hydrous minerals, such as serpentine and chlorite, are similar to the A-site in amphibole. This indicates that these hydrous minerals are candidates for the host phases of noble gases. In fact, Kendrick *et al*.^5^ measured the concentration of noble gas isotopes in metamorphic rocks and found that the signature of noble gas in serpentinites reflects that of sea water. As the breakdown of serpentine finishes at the upper part of the upper mantle (~200 km depth), it is difficult to transport to great depths in the deep mantle.

Argon is a noble gas and has three isotopes, ^36^Ar, ^38^Ar and ^40^Ar. Holland and Ballentine^6^ proposed that Ar from the mantle is identical to the seawater component using isotope analysis. This indicates that seawater recycling dominates the behaviour of Ar in the mantle. In contrast, the systematic analysis of isotopes of noble gases indicates that ocean island basalt has a primordial signature that is different from the atmospheric component^7^. The recycling of Ar between the Earth’s surface and the deep mantle has been discussed using the isotope data of noble gases. However, it is difficult to understand the mechanism of recycling of Ar because of a lack of knowledge of the physical and chemical properties of Ar at high pressures and temperatures.

Experimental and theoretical investigations of noble gases are of great interest in physics and chemistry because of their closed-shell electronic configuration and their inert nature. It is known that noble gas solids are suitable candidates for an internal pressure standard in X-ray diffraction high-pressure studies using diamond anvil cells. Noble gas solids also prove to be excellent hydrostatic pressure media in diamond anvil cell experiments. Ar becomes solidified at ~1 GPa, and it is known that Ar is a good material of the internal pressure standard for two reasons of wide range stability of the face-centred cubic structure of Ar up to at least 114 GPa^8^, and its high melting temperature compared with that of most other metals^9^. The equation of state (EOS) of Ar has been investigated by previous experimental studies^10–16^ and the melting curve of Ar has been determined up to 750 K by experiments^17^. However, reliable data at higher temperature are still not available because of the difficulties of stable heating and temperature measurement in high-pressure experiments. Recently, the first-principles calculations have been used to investigate the physical properties of materials at high pressures and temperatures. Therefore, first-principles molecular dynamics calculations used in this study have significant advantages for the high-temperature study to investigate thermophysical properties of materials.

In this study, we use density functional theory to investigate the melting temperature and thermal properties of Ar. We also perform high-pressure experiments to determine the pressure-volume relation of Ar at room temperature. The combination of high-pressure experiments and first-principles molecular dynamics calculations allows us to determine reliable physical properties, such as the EOS and melting temperature, over a wide range of pressures and temperatures.

## Experimental Results

**Figure 1:**
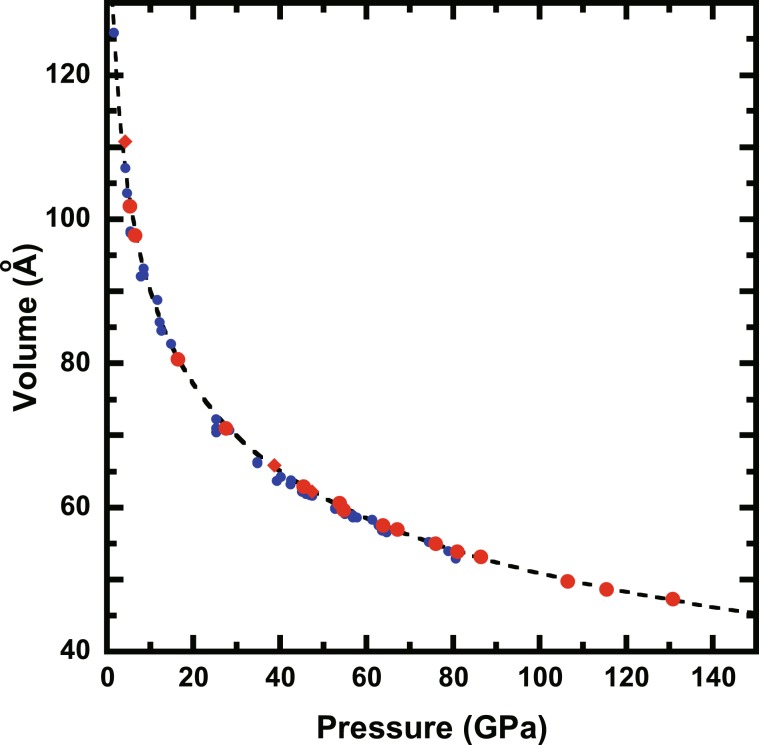
Experimental data of pressure-volume relation for Ar at 300 K. The red circles and diamonds denote the unit cell volumes of Ar with fcc structure obtained on compression and decompression, respectively. The blue circles are reported by Ross *et al*.^12^. The dashed line denotes the fitted curve using the Vinet EOS.

The volume-pressure data for Ar were obtained on compression and decompression (Supplementary Table 1). No hysteresis between compression and decompression was observed because the measurements were performed after laser-annealing to reduce the differential stress of the sample on pressure change (Fig. 1). A fit of the volume-pressure data using the Vinet EOS^18^ yielded bulk modulus values of *B*_*T*0_ = 1.07(±1.33) GPa and *B*′_*T*0_ = 8.02(±0.95), as shown in Supplementary Table 2.

The elastic parameters for Ar at room temperature have been repeatedly investigated in previous studies (Supplementary Table 3). However, a remarkable inconsistency, especially volume at ambient condition (*V*_*0*_), among previous studies has been confirmed. A possible reason for this inconsistency is that Ar is not solid at ambient condition. This indicates that the volume-pressure curve has to be extrapolated from high-pressure data to estimate the elastic parameters at ambient condition. Therefore, the elastic parameters reported by previous studies might have significant uncertainties. An advantage of our study was that the experimental condition had a wide pressure range up to 137 GPa. Our values are in general agreement with those reported by Ross *et al*.^12^.

## Computational Results

**Figure 2:**
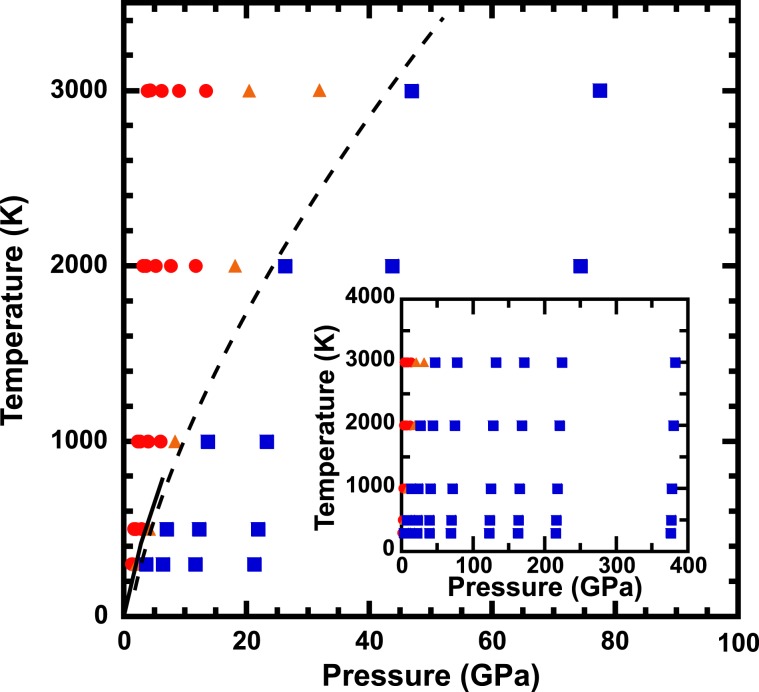
Pressure-temperature conditions where ab initio melecular dynamics (AIMD) were performed. The blue squares and red circles denote conditions where the fcc structure was stable and unstable, respectively. The orange triangles denote intermediate conditions between stable and unstable states. The dashed and solid lines denote the melting curve inferred from AIMD in this study and experimental data from Datchi *et al*.^17^. The inset shows all conditions where AIMD were performed.

Calculations were carried out at 1–382 GPa and 300–3000 K (Fig. 2). At high temperatures, the fcc structure of solid Ar was not stable (circles and triangles in Fig. 2). This indicated that liquid Ar was stable at high temperatures. The volume-pressure-temperature data of solid argon were used to analyze the EOS (squares in Fig. 2).

**Figure 3:**
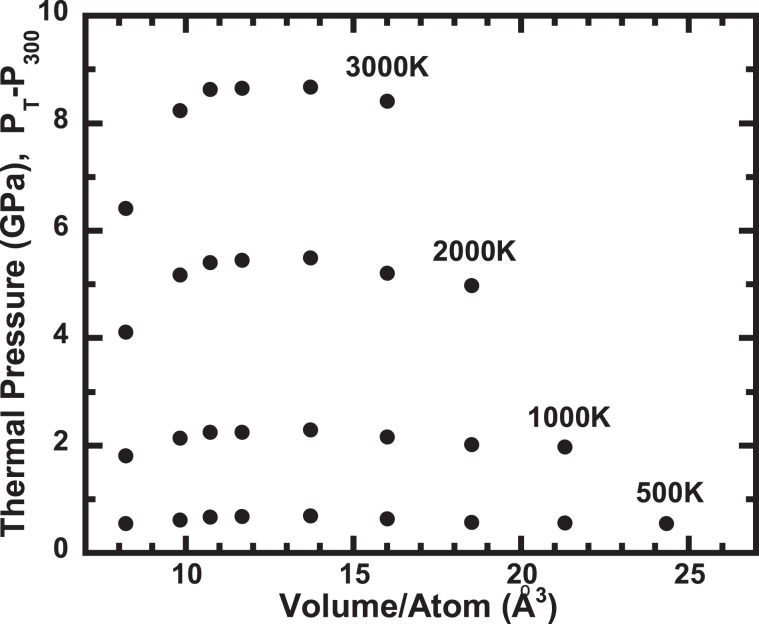
A plot of the thermal pressure calculated by AIMD. The solid circles denote the calculated thermal pressures from 500 K to 3000 K.

**Figure 4:**
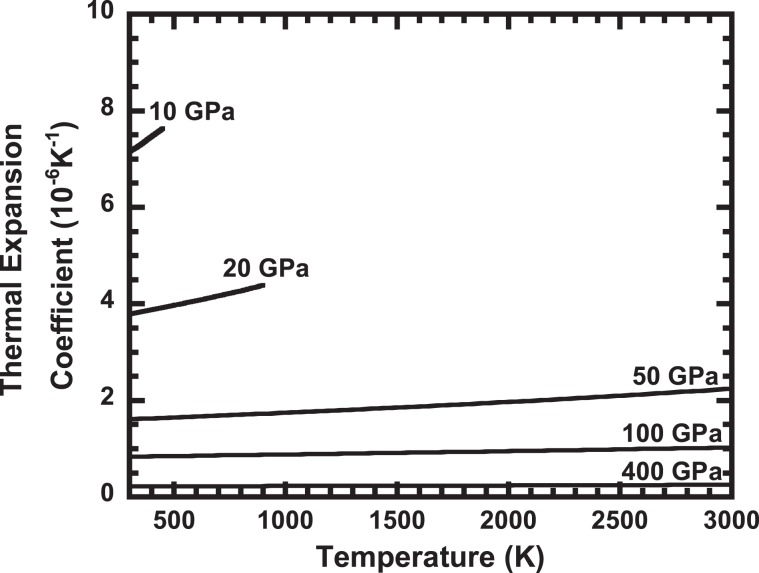
Calculated temperature dependence of the thermal expansion coefficient. The results are shown for 10, 20, 50, 100 and 400 GPa.

Figure 3 shows the relationship between thermal pressures and volumes. At low temperatures, the dependence of thermal pressure on volume was small. In contrast, the volume dependence was significant at temperatures higher than 2000 K. The significant dependence of thermal pressure on temperature indicates that the Grüneisen parameter has a large temperature dependence at higher temperatures, similar to the behaviour of metals and ionic crystals^19,20^. The calculated thermal expansion coefficient is shown in Fig. 4. The coefficient and its dependence on temperature decrease with increasing pressure, consistent with the general behaviours of solid substances.

**Figure 5:**
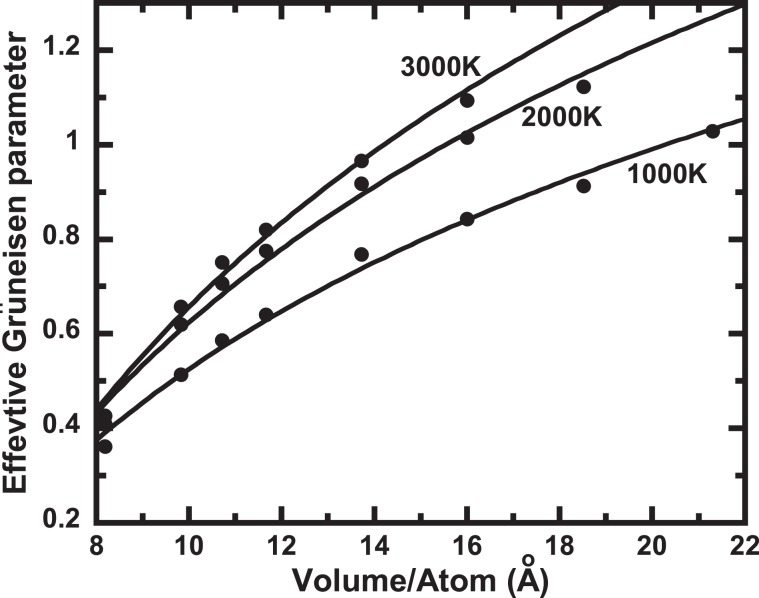
Anharmonic effects on Grüneisen parameter. The solid circles denote the calculated effective Grüneisen parameter at 1000, 2000, and 3000 K from the AIMD calculations. The solid lines denote the fitted curves using the logarithmic function.

The conventional analysis for the EOS of a solid often rules out an anharmonic effect. However, it is known that the anharmonic effect is not negligible at high temperatures. Therefore, we investigate the Grüneisen parameter to assess the anharmonic effect. The Grüneisen parameter can be obtained in our calculations from

1 $$\gamma =\frac{V{P}_{th}}{{E}_{th}}$$

where *E*_*th*_ is the difference of the internal energy. The effective Grüneisen parameter can be written as follows

2 $${\gamma }_{eff}(V,T)={\gamma }_{qh}(V)-a(V,T)$$

where *γ*_*qh*_*(V)* and *a(V, T)* are the quasiharmonic Grüneisen parameter and the intrinsic anharmonicity term, respectively. If the anharmonic effect is negligible, then the effective Grüneisen parameter does not change at high temperatures. Therefore, we calculated the effective Grüneisen parameter at different volumes and temperatures. Figure 5 shows the temperature dependence of the effective Grüneisen parameter. The anharmonic effect was significant at low pressures and decreased with increasing pressure. This indicates that the conventional analysis for the EOS, such as the quasiharmonic approximation, has a significant uncertainty in the determination of the EOS of Ar at high temperatures.

## Discussion

**Figure 6:**
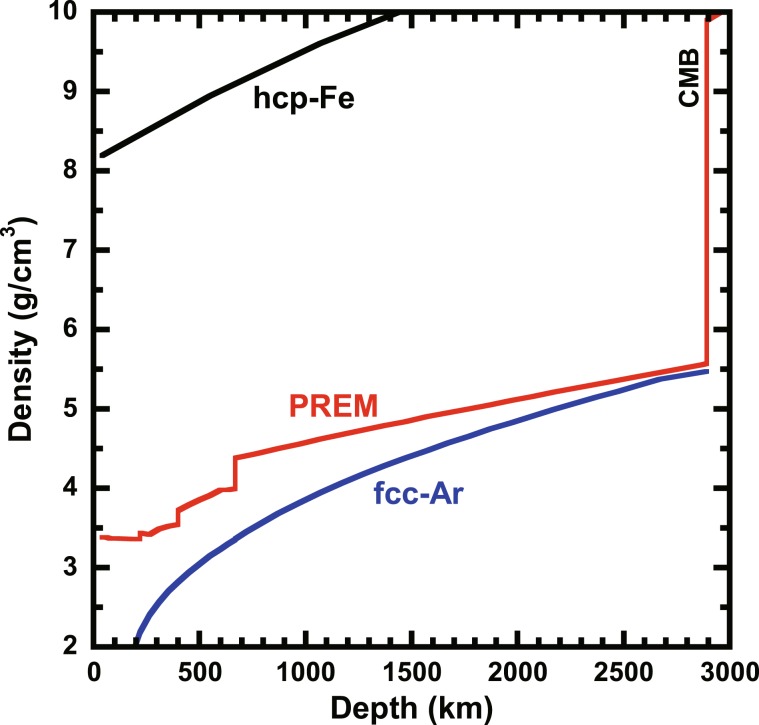
Densities of solid Ar and PREM mantle. The blue, black and red lines denote the densities of fcc-Ar, of hcp-Fe^32^ and of reference model of the mantle proposed by Dziewonski and Anderson^21^. The uncertainty of fcc-Ar is ~0.01 g/cm^3^ at depth of bottom of the lower mantle.

Figure 6 shows a comparison of densities between Ar and PREM^21^. A density difference between Ar and PREM is significant in the upper mantle and the transition zone. In contrast, the density difference decreases as the depth increases in the lower mantle. However, the density of Ar is smaller than that of PREM over the mantle condition. Jephcoat^13^ reported that the density crossover between Ar and PREM was expected at the bottom of the lower mantle, which is inconsistent with our study. This inconsistency might be due to the bulk modulus used in the previous study^13^. The value of 3.03 GPa reported in Anderson and Swenson^10^ was used to estimated the density of Ar. In contrast, recent studies reported that the value is ~1 GPa (Supplementary Table 3). Therefore, Jephcoat^13^ overestimated the density of Ar in the lower mantle.

**Figure 7:**
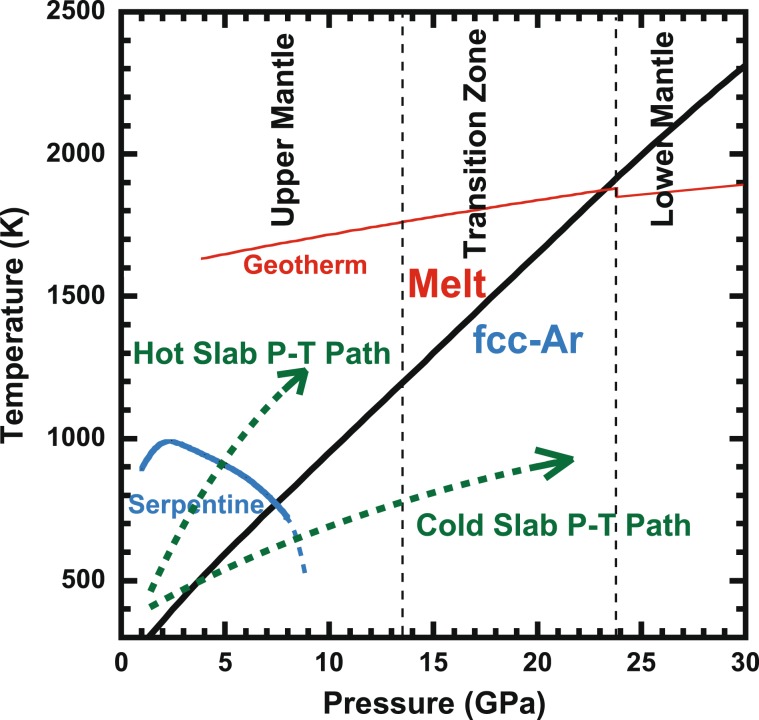
Comparison with melting curve of Ar, P-T limit of serpentine, mantle geotherm, and P-T paths of subducting slabs. Serpentine and geotherm lines are from Ulmer and Trommsdort^33^ and Ono^34^, respectively. Dashed lines are hypothetical T-P paths of cold and hot slabs.

The melting temperature of Ar increases rapidly compared with the mantle geotherm (Fig. 7). As the crossover between the melting temperature of Ar and the mantle geotherm locates at the bottom of the transition zone, Ar might be in the solid state in the lower mantle. In the case of the subducted slab, the temperature in the slab is lower than the mantle geotherm. Therefore, the stability depth of the solid Ar expands to the shallower region in the mantle. According to the relationship between the melting temperature of Ar, the slab P-T path, and the stability field of serpentine, the transport mechanism of Ar into the deep mantle is expected. A critical P-T condition is ~750 K and ~7.5 GPa, which is the crossover between the melting temperature of Ar and the stability limit of serpentine.

**Figure 8:**
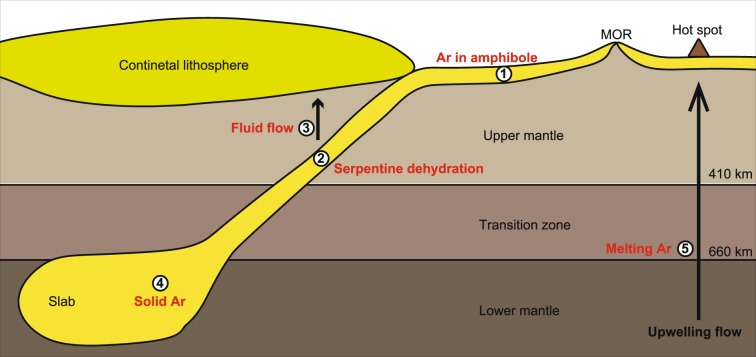
Deep argon circulation. (1) Seafloor hydrothermal circulation causes the formation of amphibole including Ar in A-sites. (2) Breakdown of serpentine yields hydrous fluid. (3) Vertical transport of liquid Ar accompanying hydrous fluid, if the slab temperature is higher than the critical P-T condition. (4) Solid Ar migrates into the deep mantle in the subducted slab, if the slab temperature is lower than the critical P-T condition. (5) Melting of solid Ar occurs in the upwelling flow at depth corresponding to a boundary between transition zone and lower mantle.

Figure 8 shows a schematic illustration of the deep argon cycle. A significant amount of Ar is trapped by hydrous minerals, such as amphibole, in the altered oceanic crust. The host phase for Ar might be serpentine at the depth of the middle upper mantle. A dehydration reaction of serpentine occurs, and most of the dehydrated fluid separates from the subducted slab. In the case of the high-temperature slab P-T path, Ar released from serpentine is liquid and escapes from the slab accompanied by the migration of dehydrated fluid. This indicates that the behaviour of Ar is an incompatible element. Then, Ar circulates in the shallower part of the upper mantle at the subduction zone. In the case of the low-temperature slab P-T path, the released Ar is solid and remains in the rock after the serpentine dehydration. The solid Ar can be transported into the deep mantle even if the slab temperature approaches the mantle geotherm, because the melting temperature of Ar is higher than the geotherm in the lower mantle (Fig. 7). After the subducted slabs circulate in the lower mantle, some of them are transported from the lower mantle into the transition zone by the upwelling mantle flow, such as under hot spots. When the upwelling flow passes across the boundary between the lower mantle and the transition zone, the solid Ar might melt and separate from the rock. If the silicate melts or aqueous fluid exists, the liquid Ar dissolves into them. Therefore, Ar cannot remain in the rock, and the transportation of Ar is controlled by the migration of melt or fluid in the transition zone and the upper mantle.

## Methods

High-pressure X-ray diffraction experiments were performed using a laser-heated diamond anvil cell high-pressure apparatus. Pure Ar gas (99.999% purity) and gold powder (99.5% purity) were used as the starting materials. First, a small amount of fine gold powder to absorb the laser radiation to provide a heat source was placed in the sample chamber, and gold was also used as an internal pressure calibrant. Next, Ar was cryogenically loaded into the sample chamber. The starting material was compressed at room temperature with symmetrical or motor-driven diamond anvil cells^22,23^. As the differential stress during room temperature compression causes a significant systematic bias for the relationship between pressure and structural properties, the samples were heated after each change in pressure using an infrared laser to reduce any differential stress in the sample (Supplementary Fig. S1). The temperature and duration of the annealing were 1000–2000 K and 3–5 minute, respectively. The samples were probed using an angle-dispersive X-ray powder diffraction technique at the synchrotron beam lines AR-NE1A at the Photon Factory or BL10XU at SPring-8. Experimental assemblies for synchrotron X-ray measurements have been described elsewhere^24,25^. A monochromatic incident X-ray beam was used in both synchrotron beam lines. The X-ray beams were collimated to a diameter of 20–30 µm at pressures lower than 60 GPa, and <10 µm at higher pressures. We monitor the X-ray beam intensity distribution transmitted through the DAC by scanning the DAC stage to adjust the sample position to the X-ray beam position precisely. The image of the sample and the gasket hole are reflected in the obtained two-dimensional map of the transmitted X-ray intensity. This X-ray map was used to set the sample on the X-ray beam position. The angle-dispersive X-ray diffraction patterns were obtained on imaging plates. The observed intensities on the imaging plates were integrated as a function of 2θ to obtain conventional, one-dimensional diffraction profiles. In order to determine the unit-cell volumes of Ar and gold, 3–5 lines (111), (200), (220), (311), and (222) were used for the calculation. Pressures were determined from the unit-cell volumes of the gold powder in the diamond anvil cell, using the EOS reported by Dorogokupets and Dewaele^26^. EOS parameters for solid Ar were obtained by a least-square fit to the pressure-volume data of the Vinet EOS^18^.

The first-principles calculations carried out in this study were based on density functional theory using the Vienna Ab initio Simulation Package (VASP)^27^. For the exchange-correlation potential, the PBE-sol functional was used in the generalised gradient approximation (GGA) calculations^28^. The electronic wave functions were expanded in a plane-wave basis set with a cut-off energy of 600 eV, and the electron-ion interactions were described using the projector augmented wave (PAW) method. The PAW potential of Ar had the outermost cutoff radii of the valence orbital of 0.953 Å, with a 3 s^2^ 3p^6^ valence configuration. We used a 108-atom supercell with gamma-point Brillouin zone sampling and a time step of 1 fs for the first-principles molecular dynamics simulation at constant volume. Simulations were run in the constant NVT ensemble with the Nosé thermostat^29^ 5–10 ps after equilibration. Details of our methodology have been given elsewhere^30^. The computation time required to reach equilibration varied between configurations, and depended on the starting atomic positions, velocity, temperature, and pressure. The first-principles molecular dynamics calculations were performed under 65 pressure-volume conditions in this study. The pressure and temperature ranges were 1–382 GPa and 300–3000 K, respectively. The thermal pressure was calculated at each volume. The total pressure at high temperatures and pressures conditions was estimated from room-temperature EOS from experimental data and the thermal pressure from the first-principles molecular dynamics calculations. It is known that the density functional theory has a few % uncertainty depended on the approximation^30^. Therefore, the values of the thermal pressure calculated in this study has the uncertainty of ~few %.

### Analysis

The pressures of solids can be described by:

3 $$P(V,T)={P}_{st}(V,300)+{P}_{th}(V,T)$$

Where *P(V*, *T)* is the total pressure *P* at volume *V* and temperature *T*. The first and second terms on the right side of the equation represent the relationship between pressure and volume at 300 K, and the thermal pressure at volume *V*, respectively. In this study, the Vinet EOS^18^ is used for the first term of Eq. (3):

4 $${P}_{st}(V,300)=3{B}_{T0}\frac{\left[1-{\left(\frac{V}{{V}_{0}}\right)}^{\frac{1}{3}}\right]}{{\left(\frac{V}{{V}_{0}}\right)}^{\frac{2}{3}}}{{exp}}\left\{\frac{3}{2}({B^{\prime} }_{T0}-1)\left[1-{\left(\frac{V}{{V}_{0}}\right)}^{\frac{1}{3}}\right]\right\}$$

where *B*_*T0*_ is the isothermal bulk modulus, and *B*′_*T0*_ is ($\partial {B}_{T}/\partial P$)_*T*_ at ambient temperature. In the thermal pressure EOS^31^, *P*_*th*_, can be written as follows:

5 $${P}_{th}(V,T)={\alpha }_{0}{B}_{T0}(T-{T}_{300})+{\left(\frac{\partial {B}_{T}}{\partial T}\right)}_{V}(T-{T}_{300}){ln}\left(\frac{{V}_{0}}{V}\right)+{\left(\frac{{\partial }^{2}P}{\partial {T}^{2}}\right)}_{V}{(T-{T}_{300})}^{2}$$

where *α*_*0*_, ($\partial {B}_{T0}/\partial T$)_*V*_, and (${\partial }^{2}P/\partial {T}^{2}$)_*V*_ are the coefficient of the volume thermal expansion at ambient condition, the temperature derivative of the isothermal bulk modulus at constant volume, and the second temperature derivative of the pressure at constant volume, respectively. Finally, Eq. (3) is expressed as

6 $$\begin{array}{rcl}P(V,T) & = & 3{B}_{T0}\frac{\left[1-{\left(\frac{V}{{V}_{0}}\right)}^{\frac{1}{3}}\right]}{{\left(\frac{V}{{V}_{0}}\right)}^{\frac{2}{3}}}{{exp}}\left\{\frac{3}{2}({B^{\prime} }_{T0}-1)\left[1-{\left(\frac{V}{{V}_{0}}\right)}^{\frac{1}{3}}\right]\right\}\\  &  & +\,\left[{\alpha }_{0}{B}_{T0}({V}_{0})+{\left(\frac{\partial {B}_{T0}}{\partial T}\right)}_{V}\,{ln}\left(\frac{{V}_{0}}{V}\right)+{\left(\frac{{\partial }^{2}P}{\partial {T}^{2}}\right)}_{V}(T-300)\right](T-300)\end{array}$$

Equation (4) was used to fit the pressure-volume-temperature data from our experiments and calculations. The typical uncertainty of calculated pressure including errors from the high-pressure experiments and the first-principles calculations is less than 1 GPa around 100 GPa.

## Supplementary information

Supplementary Information
