# Supplementary material for: Fate of subducted argon in the deep mantle
Source: Sci Rep. 2020 Feb 3;10:1393. doi: 10.1038/s41598-020-58252-8 (PMC6997185; doi:10.1038/s41598-020-58252-8)
Supplement: Supplementary file 1 — Supplementary Information [file 41598_2020_58252_MOESM1_ESM.pdf]

Supplementary files

Title: Fate of subducted argon in the deep mantle

Author: Shigeaki Ono

Volcanoes and Earth's Interior Research Center, Research Institute for Marine Geodynamics, Japan Agency for Marine-Earth Science and Technology, 2-15 Natsushima-cho, Yokosuka, Kanagawa 237-0061, Japan.

Table 1. Pressure-volume data of Ar up to 137 GPa at room temperature.

P (GPa)	Volume (Å^3)	P (GPa)	Volume (Å^3)
4.44(5)	110.74(23)	56.9(5)	59.64(12)
5.41(6)	101.82(17)	66.3(4)	57.52(14)
6.67(5)	97.81(22)	69.9(1)	56.96(14)
16.6(1)	81.22(6)	79.1(1)	55.04(9)
16.9(1)	80.56(2)	84.4(2)	53.88(10)
28.4(1)	70.99(9)	90.1(2)	53.16(16)
40.1(1)	65.84(4)	111.2(6)	49.79(3)
47.1(2)	62.90(6)	120.6(4)	48.68(4)
49.1(2)	62.17(14)	136.7(3)	47.26(10)
55.9(5)	60.60(7)		

X-ray diffraction data were measured at room temperature after annealing. The numbers in parentheses are the error (standard deviation, 1σ).

Table 2. Thermoelastic parameters of Ar.

Parameter	
Experiment	
V_0 (Å^3)	184.5(38.5)
B_{T0} (GPa)	1.07(1.33)
B'_{T0}	8.02(0.95)
AIMD	
αB_T (GPa/K)	0.0038
$(\partial B_T/\partial T)_V$ (GPa/K)	-0.00065
$(\partial^2 B_T/\partial T^2)_V$ (10^{-8}GPa^2/K^2)	5.2

Table 3. Comparison of elastic parameters of Ar at room temperature.

B_{T0} (GPa)	B_T'	V_0 (Å^3)	
3.03[a)]	7.24[a)]	149.83[a)]	Anderson & Swenson[10]
0.99(1.59)	8.08(1.3)	182.8(46.9)	Ross et al.[12]
6.5(5)	5.1(3)	143(11)	Errandonea et al.[8]
1.66	8.3	175	Chen et al.[15]
0.44	8.92	203.1	Marquardt et al.[16]
1.07(1.33)	8.02(0.95)	184.5(38.5)	This study

[a)]Values were determined at 7 K. The numbers in parentheses are the error.

Ono
Fig. S1

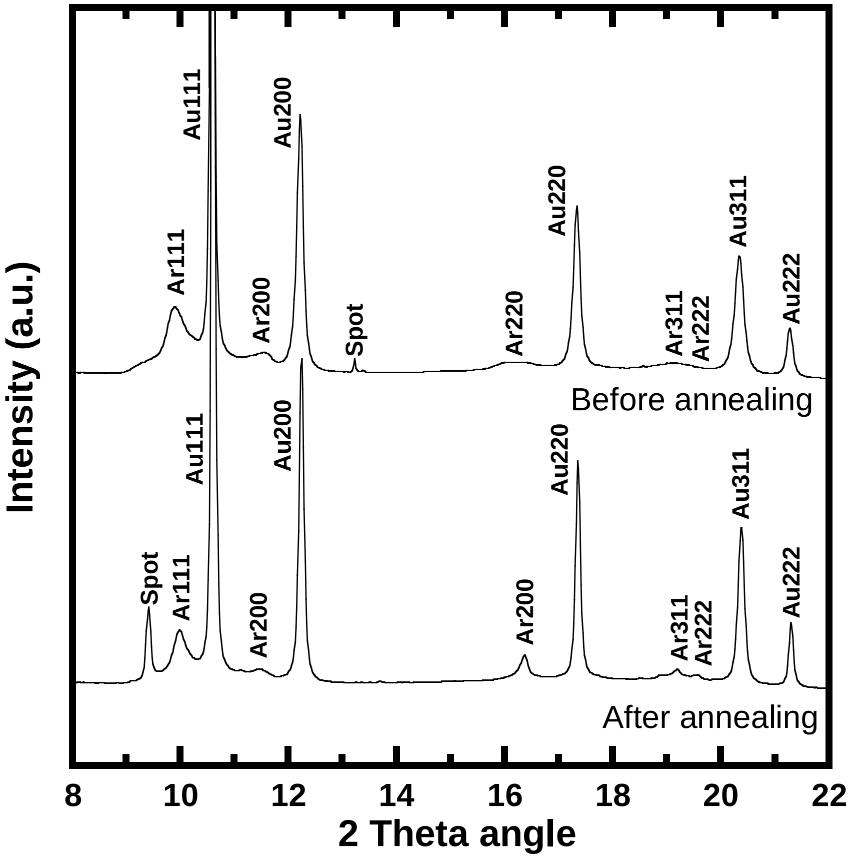

Fig. S1.

Comparison of powder X-ray diffraction data between before and after annealing. Upper and lower data denote before and after annealing. The labels for the diffraction peaks are as follows: Ar – argon with the fcc structure and Au – gold. The numbers on labels correspond to the indexes of the cubic symmetry. The wavelength of the monochromatic incident X-ray beam was $\lambda = 0.4177$Å.
